# Lipopolysaccharide-induced cytokine signaling activates a temporal innate defense program and represses pancreatic β-cell identity

**DOI:** 10.1016/j.jbc.2025.110811

**Published:** 2025-10-13

**Authors:** Jacob T. Bartosiak, Polly A. Hansen, Elaine A. Schumacher, Katherine R. Harty, Jennifer S. Stancill, John A. Corbett

**Affiliations:** Department of Biochemistry, Medical College of Wisconsin, Milwaukee, Wisconsin, USA

**Keywords:** β-cell, interleukin 1, islet, PAMP, antiviral

## Abstract

Cytokine exposure has been promoted as one cause for the loss of β-cell mass during the development of type 1 diabetes. While *in vitro* studies have shown that cytokines inhibit insulin secretion and induce islet degeneration, interventions targeting these soluble mediators have had limited success in preventing disease development in rodents and humans. To understand the *in vivo* actions of cytokines in the endocrine pancreas, we explored the transcriptional responses of islets to endogenously produced cytokines following immune stimulation with the bacterial pathogen-associated molecular pattern lipopolysaccharide. We found that endogenous cytokine production in response to lipopolysaccharide administration stimulates the rapid, time-dependent expression of antiviral, antibacterial, and antioxidant genes and represses the expression of β-cell identity factors in islets. Changes in gene expression are associated with similar changes in protein expression and the actions are transient, with a return to control levels of gene expression 24 h post lipopolysaccharide administration. In contrast to a role in diabetes development, our findings support a physiologically relevant and dynamic immune-endocrine communication axis that is characterized by a cytokine-initiated cell-intrinsic defense response in the endocrine pancreas that has evolved to enhance the fitness of these essential cells during host infection.

Islets of Langerhans are highly vascularized micro-organs of the endocrine pancreas that regulate glucose homeostasis by releasing endocrine hormones. In response to elevated glucose, β-cells release insulin to stimulate glucose disposal in peripheral tissues while glucagon, produced by α-cells, stimulates hepatic glucose output to prevent hypoglycemia between meals. While β-cells are indispensable for survival as the sole cellular source of insulin, they are terminally differentiated with minimal proliferative capacity. Consequently, the loss of functional β-cell mass, as in type 1 diabetes (T1D), requires lifelong exogenous insulin treatment.

Based on studies performed over the past 40 years, it is generally believed that cytokines, such as interleukin-1 (IL-1), contribute to the development of T1D by inhibiting insulin secretion and contributing to β-cell death (1). Alone, or in combination with other inflammatory cytokines (tumor necrosis factor (TNF); interferon-γ(IFN-γ)), treatment of isolated rodent and human islets with IL-1 results in an inhibition of mitochondrial oxidative metabolism and insulin secretion and decreased islet cell viability (2, 3). Nitric oxide, produced following the induction of inducible nitric oxide synthase (iNOS), mediates these actions of cytokines by inhibiting the Krebs cycle enzyme aconitase and complex IV of the electron transport chain, resulting in a decrease in ATP (4). We and others have shown that islets treated with inhibitors of iNOS (5, 6, 7) and islets isolated from iNOS-deficient mice (8) are resistant to the inhibitory and destructive actions of cytokines.

While not generally acknowledged, the actions of cytokines and nitric oxide on mitochondrial metabolism and insulin secretion are reversible either by removal of the cytokine or inhibition of nitric oxide production (9, 10, 11) suggesting that cytokine signaling in islets may have physiological relevance. Further, we did not observe an increase in the accumulation of proapoptotic genes by single-cell RNA sequencing (scRNA-seq) analysis of mouse and human islets treated for 6 h and 18 h with cytokines (12, 13, 14). In fact, antiviral and antibacterial (antipathogen) genes were among the most highly upregulated genes in response to cytokine treatment (12).

Given that hypotheses concerning the role of inflammatory cytokines in diabetes development are predominantly based on *in vitro* studies using isolated islets and purified populations of endocrine cells, the goal of this study was to evaluate the effects of endogenously produced cytokines on gene expression in endocrine cells. We chose a well-characterized, nonseptic dose of lipopolysaccharide (LPS) that, when delivered IP, stimulates systemic cytokine production associated with mild-transient illness behavior (hypoglycemia and reduced social behavior) (15, 16) that returns to baseline ∼24 h post administration (15). We show that there is a time-dependent increase in the expression of antiviral, antibacterial, and antioxidant genes and repression of genes associated with β-cell identity in islets harvested from LPS treated mice. These changes in expression are transient, returning to levels observed in saline treated control mice 24 h post injection. Further, the changes in islet gene expression are not the result of direct LPS signaling in the endocrine compartment but rather systemic cytokine production in response to this endotoxin. The dynamic interaction between the immune and endocrine systems observed in this study supports our hypothesis that soluble mediators produced by the innate immune system serve as alarm signals of potential danger and function to initiate cell-intrinsic defense mechanisms to enhance survival of these essential cells.

## Results

### Activation of antipathogen gene expression in cytokine treated islets

**Figure 1:**
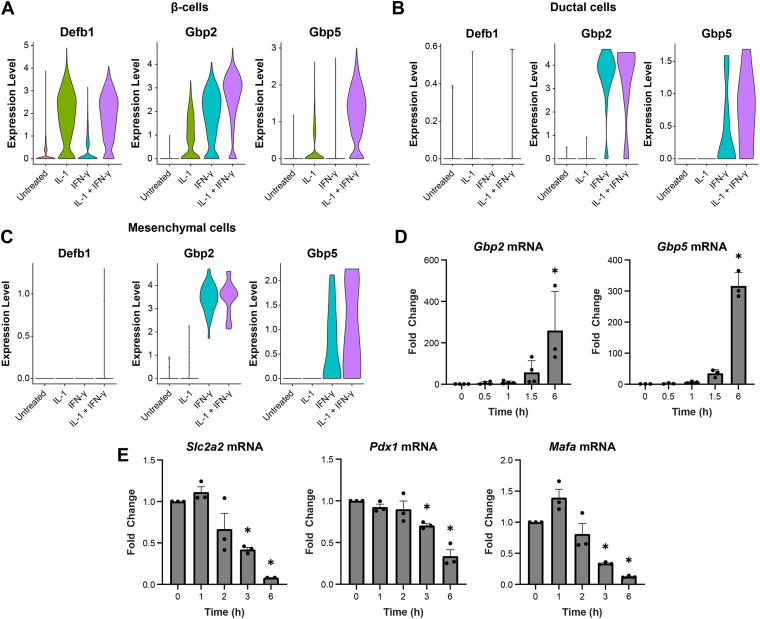
**Time-dependent effects of IL-1 on gene expression in islets.***A*-*C*, islets from C57BL/6J mice were incubated with cytokines (10 U/ml IL-1; 150 U/ml IFN-γ) for 6 h. Gene expression in β-cells, ductal cells, and mesenchymal cells, assessed by single-cell RNA sequencing, is shown. *D*-*E*, islets were incubated for the indicated times with IL-1 (10 U/ml) and changes in mRNA accumulation of the indicated genes were determined by qRT-PCR. Results are the average ± SD of three independent experiments. Statistical significance calculated with one-way ANOVA (∗*p* < 0.05 *versus* untreated control; ns = not significant) is indicated. IFN-γ, interferon-γ.

As previously described, scRNA-sequencing of isolated mouse islets treated for 6 h with IL-1 and IFN-γ revealed an increase in endocrine cell expression of genes that participate in antiviral and antibacterial defense (12). In further analyzing these data, IL-1 stimulated antiviral defense responses in endocrine cells, but failed to do so in non-endocrine cells (Fig. 1, *A*–*C*). The lack of an IL-1 response in ductal and mesenchymal cells was not due to lack of expression of the IL-1 receptor (12). These results suggest that one of the initial responses of the endocrine pancreas to IL-1 is the induction of protective and anti-pathogen gene expression. Further, the response is rapid; within 60 min of IL-1 treatment of islets isolated from C57BL/6J mice, there is a greater than 5-fold increase in the accumulation of guanylate binding protein 2 (*Gbp2*) and guanylate binding protein 5 (*Gbp5*) mRNA, as determined by quantitative PCR (Fig. 1*D*). Following a 6 h exposure, *Gbp2* and *Gbp5* mRNA levels increase by greater than 200-fold. While β-cells increase antipathogen gene expression in response to IL-1, there is also a rapid repression of mRNAs encoding β-cell identity genes including *Mafa*, *Slc2a2* (GLUT2), and *Pdx1* (Fig. 1*E*).

### Low dose LPS administration stimulates soluble mediator production but does not directly modify gene expression or signaling in β-cells

**Figure 2:**
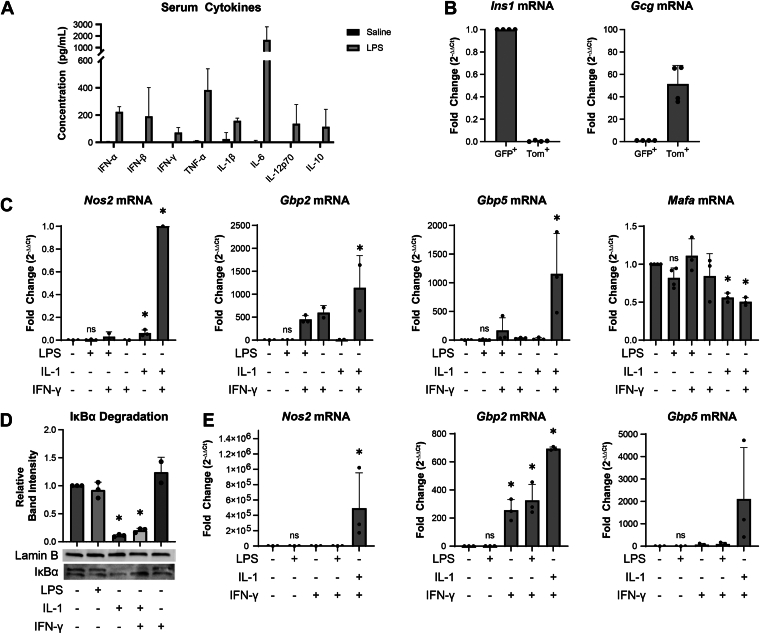
**Effects of LPS onserum cytokines andgene expression.***A*, analysis of serum cytokine levels from C57BL/6J mice 6 h after intraperitoneal administration of lipopolysaccharide (LPS) (0.33 mg/kg) or endotoxin-free physiological saline (*n* = 4 mice per group). *B*, hormone gene expression in FACS-purified islet cell populations enriched for β-cells (GFP^+^) or islet-non-β-cells (Tom^+^). Effects of LPS (20 μg/ml), IL-1 (10 U/ml), and IFN-γ (150 U/ml) on *Nos2,Gbp2,Gbp5,* and *Mafa* mRNA accumulation in FACS-purified mouse β-cells after 6 h incubation as determined by qRT-PCR (*C*), on IκBα levels in mouse islets treated for 30 min as measured by Western blot analysis (*D*), and on the levels of *Nos2, Gbp2,* and *Gbp5* mRNA in INS832/13 cells following a 6 h treatment as evaluated by qRT-PCR (*E*). Results are the average ± SD of 3 to 4 independent experiments (*A*-*C*, *E*) or representative of 3 independent (*D*) experiments. Statistical significance calculated by one-way ANOVA (∗*p* < 0.05 *versus* untreated control; ns = not significant) is indicated. FACS, fluorescence-activated cell sorting; Gbp2, guanylate binding protein 2; Gbp5, guanylate binding protein 5; LPS, lipopolysaccharide; IFN-γ, interferon-γ.

LPS is a well-characterized bacteria-derived pathogen-associated molecular pattern (PAMP) that activates the innate immune system and, when administered at high doses, is used as a model of sepsis (17). At low doses, LPS activates the innate immune system with peak increases in serum cytokine levels and mild transient illness behavior – characterized by temporary reduction in social behavior and reduced blood glucose – observed within 4 to 8 h of administration (15, 16). At these doses, LPS-stimulated events return to normal 24 h post administration (15). Consistent with these studies (17, 18), there is an increase in serum levels of IFN-α, IFN-β, IFN-γ, TNF-α, IL-1β, IL-6, IL-12p70, and IL-10 6 h post administration of 0.33 mg/kg LPS by IP injection in C57BL/6J mice (Fig. 2*A*). These cytokine levels are consistent with those observed in mice following coxsackievirus B3 infection (19).

LPS was chosen as an immune agonist because its modes of action are well-defined, and it does not directly alter gene or protein expression in β-cells. In support of the latter statement, we have shown that LPS activates resident macrophages to produce IL-1 locally in islets to levels sufficient to stimulate iNOS expression and nitric oxide production, and to inhibit insulin secretion in rat and human islets (20, 21, 22). However, LPS does not modify iNOS expression in fluorescence-activated cell sorting (FACS) purified rat β-cells (20). To confirm these findings, β-cells were purified by FACS from *Ins1*^*Cre/+*^*; ROSA26*^*mTmG/+*^ mouse islets where β-cells express green fluorescent protein (GFP) and non-β-cells express tdTomato. Hormone expression in the purified GFP^+^ cell populations, assessed by qRT-PCR, was used to confirm enrichment of β-cells and non-β-cells (Fig. 2*B*). Consistent with observations in rat β-cells, LPS (6 h treatment) fails to stimulate the expression of *Gbp2*, *Gbp5*, or *Nos2* (iNOS)*,* or to repress the expression of the β-cell identity gene *Mafa* in FACS-purified mouse β-cells (Fig. 2*C*). LPS also does not activate NF-κB in C57BL/6 islets as assessed by IκBα degradation 30 min after LPS stimulation (Fig. 2*D*), nor does it stimulate *Nos2*, *Gbp2*, or *Gbp5* expression by rat insulinoma INS832/13 cells (Fig. 2*E*). As a positive control, the actions of IL-1 and IL-1+ IFN-γ on gene expression and IκBα degradation are shown.

### The response of the endocrine pancreas to innate immune activation

**Figure 3:**
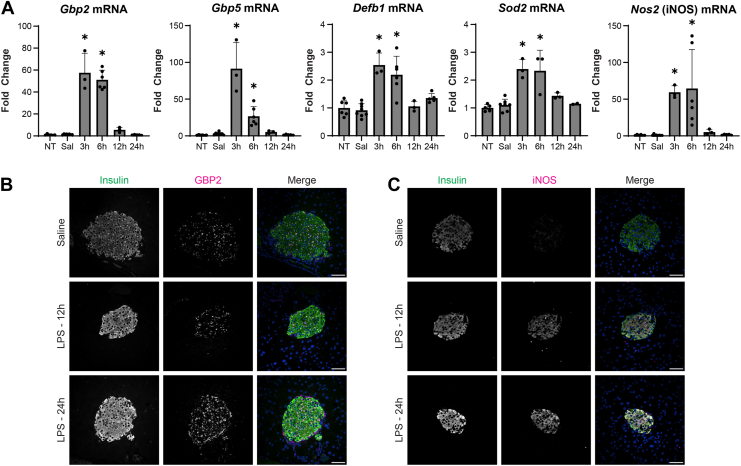
**Islet gene and protein expression following LPS administration to mice.***A*, relative gene expression in islets isolated from C57BL/6J mice at the indicated times after IP LPS (0.33 mg/kg) or endotoxin-free physiological saline administration as determined by qRT-PCR (n = 3–8 mice per group). Results are the average ± SD. Statistical significance calculated by one-way ANOVA (∗*p* < 0.05 *versus* untreated control; ns = not significant) is indicated. *B*, representative images from pancreatic sections (average of N = 23 islets per condition from 3 replicates) from mice administered saline or LPS (12 h or 24 h) and probed for insulin and GBP2 or (*C*) inducible nitric oxide synthase. Nuclei are marked with DAPI (*blue*). Scale bar = 50 μm. Gbp2, guanylate binding protein 2; LPS, lipopolysaccharide.

After confirming that LPS does not directly modify gene expression in β-cells, the effect of low dose (0.33 mg/kg) LPS administration (IP) on gene expression in islets was evaluated in C57BL/6J mice 3 to 24 h post treatment. Islet expression of the antiviral genes *Gbp2* and *Gbp5*, antibacterial gene beta-defensin 1 (*Defb1*), antioxidant gene superoxide dismutase 2 (*Sod2*), and *Nos2* were increased 3-6 h post LPS administration (Fig. 3*A*). Expression of each of these genes returns to baseline levels 24 h post LPS administration. The induction of a transient transcriptional response in the pancreatic islet following *in vivo* administration of LPS is consistent with the response observed following a 6 h *in vitro* incubation of islets with IL-1(12). Importantly, changes in islet gene expression return to baseline levels 24 h after LPS administration.

Protein expression of GBP2 (Fig. 3*B*) and iNOS (Fig. 3*C*) was evaluated by immunofluorescence microscopy in pancreas sections collected from mice 12 or 24 h after LPS treatment to determine if changes in gene expression are reflected at the protein level. 24 h post LPS administration there is an increase in the expression of GBP2 and iNOS (magenta) in insulin-containing cells (green) (quantified in Fig. S1, *A* and *B*), indicating islet transcriptional responses observed in response to LPS administration correlate with changes in protein expression and colocalize with insulin (Fig. 3, *B* and *C*). These islet responses do not appear to promote immune cell infiltration or insulitis as assessed by hematoxylin and eosin-stained sections 12–24 h post LPS administration (Fig. S2).

### Immune activation transiently represses the expression of β-cell identity genes

**Figure 4:**
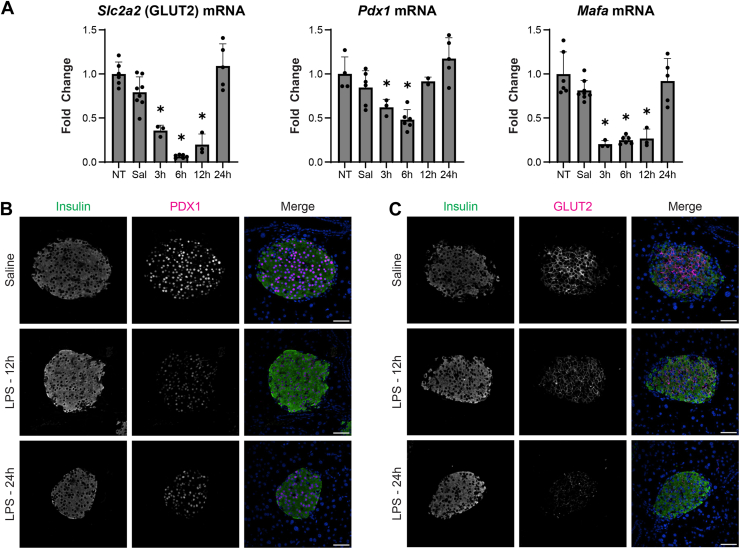
**Effects of LPS administration to mice on islet identity factor expression.***A*, mRNA accumulation of the indicated genes was determined by qRT-PCR from islets isolated from C57BL/6J mice at the indicated times after IP LPS (0.33 mg/kg) or endotoxin-free physiological saline administration (3–8 mice per group). *B*, representative images from pancreatic sections (average of N = 23 islets per condition from 3 replicates) from mice administered saline or LPS (12 h or 24 h) and probed for insulin and PDX1 or (*C*) GLUT2. Nuclei are marked with DAPI (*blue*). Scale bar = 50 μm. Results are the average ± SD. Statistical significance calculated by one-way ANOVA (∗*p* < 0.05 *versus* untreated control; ns = not significant) is indicated. LPS, lipopolysaccharide.

Decreases in the expression of β-cell identity genes have been shown to contribute to dedifferentiation and defects in insulin secretion (23). Since the repression of identity gene expression occurs within hours of cytokine exposure, we hypothesize that it may be a primary adaptive response to innate immune activation rather than a mediator of β-cell dysfunction (12). To test this hypothesis, the expression of β-cell identity genes was measured in islets 3 to 24 h post LPS administration. Consistent with the effects of IL-1 *in vitro*, steady state mRNA levels of *Mafa*, *Slc2a2* (GLUT2), and *Pdx1* were decreased within 3 to 6 h of *in vivo* LPS administration (Fig. 4*A*). We also show that the loss of mRNA correlates with a decrease in the levels of PDX1 and GLUT2 in insulin containing β-cells as assessed by immunofluorescence of pancreatic sections prepared 12 to 24 h after LPS administration (Fig. 4, *B* and *C*). Consistent with the loss of *Pdx1* and *Slc2a2* mRNA expression, PDX1 and GLUT2 protein expression is decreased 12 to 24 h after LPS administration (quantified in Fig. S1, *C* and *D*). The rapid gene responses and reversibility of β-cell identity gene repression in response to *in vivo* LPS administration suggest that these changes may represent physiologically relevant immune-endocrine signaling events.

### Insulin secretion is enhanced in mice following LPS administration

**Figure 5:**
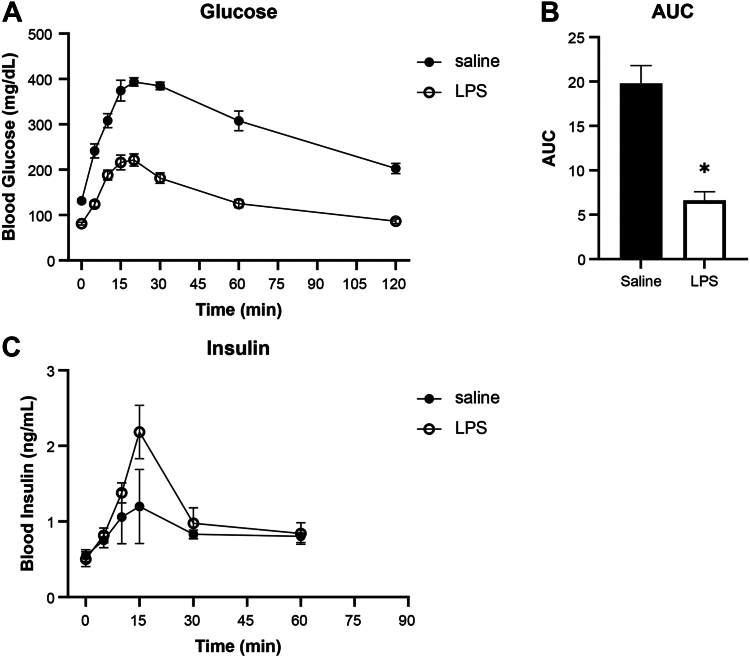
**Effects of LPS on oral glucose tolerance.***A*, blood glucose was measured during oral glucose tolerance test (2 g glucose/kg body weight) in C57BL/6J mice 24 h after IP saline or LPS (0.33 mg/kg) administration. *B*, area under the curve (AUC; mg/dl·min·10^−3^) of glucose response. *C*, blood insulin levels were determined. Results are average ± SEM of 6 mice per condition. Statistical significance calculated by Welch’s *t* test (∗*p* < 0.05).

To determine whether endotoxin alters β-cell function *in vivo*, we performed an oral glucose tolerance test (2 g/kg body weight) 24 h after LPS (0.33 mg/kg) was administered to C57BL/6J mice by IP injection (Fig. 5, *A*–*C*). Compared to mice injected with saline, LPS administration improved glucose tolerance and enhanced insulin secretion in response to the glucose challenge. While these findings are consistent with previous reports that insulin secretion is increased following LPS administration in rodents (24, 25, 26), the enhancement in β-cell function following LPS treatment paradoxically occurs at the same time protein expression of several known markers of β-cell identity is reduced.

### IL-1 is a primary soluble mediator of an immune-endocrine signaling axis

**Figure 6:**
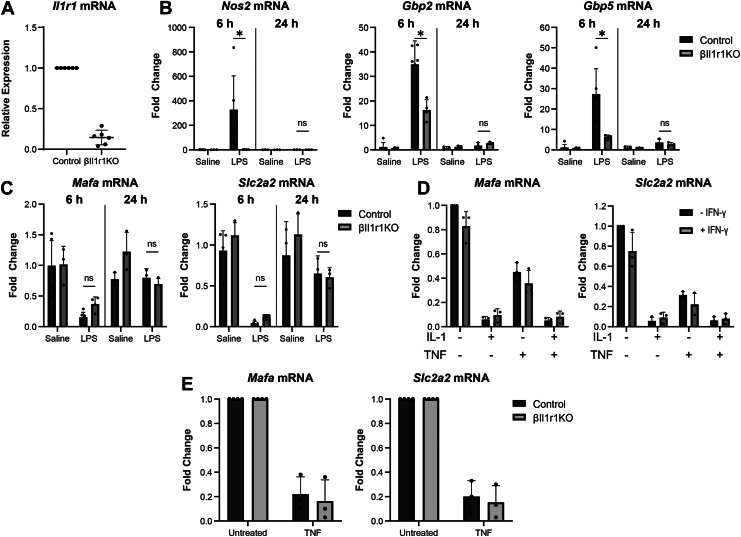
**Effects of LPS administration on islet gene expression from mice with β-cell-specific deletion of Il1r1.***A*, expression of Il1r1 mRNA in islets isolated from βIl1r1KO (Il1r11^fl/fl^; Ins1^Cre/+^) and littermate control animals (Il1r11^fl/fl^; Ins1^+/+^). *B*-*C*, gene expression in islets isolated from βIl1r1KO and littermate control animals 6 h or 24 h (n = 3–6 animals per group) post LPS (0.33 mg/kg) (IP administration) or endotoxin-free physiological saline. *D*, effects of a 6 h *in vitro* treatment with IL-1 (10 U/m), TNF (50 ng/ml) and IFN-γ (150 U/ml) on the expression of the indicated genes in islets isolated from C57BL/6J mice. *E*, effects of an 18 h incubation with tumor necrosis factor (50 ng/ml) on identity gene expression in islets isolated from βIl1r1KO or littermate control mice. Results are average ± SD of 3 to 8 independent experiments. Statistical significance calculated by two-way ANOVA (∗*p* < 0.05 *versus* untreated control; ns = not significant) is indicated. IFN-γ, interferon-γ; TNF, tumor necrosis factor.

*In vitro* studies performed over the past 4 decades have shown that β-cells are highly responsive to IL-1 leading to inhibition of insulin secretion, decreased cell viability, induction of cellular stress, and recently, the stimulation of antipathogen defense pathways (2, 12, 13, 27, 28). To directly evaluate whether IL-1 is a primary mediator of changes in endocrine cell gene expression in response to LPS administration, we generated a mouse model harboring a β-cell-specific deletion of the IL-1 signaling receptor type 1 (Il1r1). Il1r1^flox/flox^ mice (29) were bred to mice expressing Cre in the endogenous Ins1 locus (Ins1^Cre/+^) (30) to obtain Il1r1^flox/flox^; Ins1^Cre/+^ (βIl1r1KO) animals. Genomic DNA from Il1r1^flox/flox^; Ins1^Cre/+^ (βIl1r1KO) animals was observed at 379 bp compared to 420 bp in littermate control animals indicating successful excision of exons 3 and 4 of the Il1r1 gene (Fig. S3*A*) (29). Il1r1 mRNA expression in islets isolated from these animals is reduced by approximately 80% (Fig. 6*A*), consistent with β-cells accounting for the majority, but not all, of the IL1r1 expressing cells found in murine islets. As expected for mice with defective IL-1 signaling in β-cells, the stimulatory actions of IL-1 and IFN-γ on iNOS mRNA and protein expression are attenuated and nitrite accumulation is reduced in islets isolated from βIl1r1KO animals (Fig. S3, *B*–*E*). These findings are consistent with recent reports showing that multiple endocrine cell types, in addition to β-cells, express iNOS in response to this cytokine combination in murine islets (12, 13). As expected, IL-1 and IFN-γ stimulated iNOS mRNA accumulation in islets isolated from βIl1r1KO mice is completely prevented by the IL-1 receptor antagonist protein (IL-1RA) (Fig. S3*E*). Similarly, iNOS expression, evaluated by immunofluorescence, was observed in β-cells treated with IL-1 and IFN-γ for 40 h isolated from littermate control animals, but not in β-cells from βIl1r1KO islets (Fig. S3*F*).

To directly evaluate whether IL-1 is responsible for the changes in islet gene expression in response to innate immune activation, LPS (0.33 mg/kg) was administered to βIl1r1KO mice by IP injection and gene expression was evaluated by qRT-PCR in islets harvested 6 h post administration. In support of IL-1 as a primary mediator of changes in islet cell gene expression following LPS administration, the mRNA levels of *Nos2*, a canonically IL-1-dependent gene, are increased in control animals, while they were unchanged in islets isolated from mice lacking the Il1R1 in β-cells (Fig. 6*B*). The accumulation of *Gbp2* and *Gbp5* mRNA is also attenuated in βIl1r1KO mice, consistent with *Gbp2* and *Gbp5* dependence on both IL-1 and interferon signaling (Fig. 6*B*). Overall, these findings support IL-1 as one mediator of the changes in gene expression in islets observed following immune stimulation by LPS administration in mice.

While IL-1 represses β-cell identity genes in cultured islets, the levels of *Mafa* and *Slc2a2* are similarly repressed in islets isolated from βIl1r1KO or littermate control mice treated with LPS *in vivo* (Fig. 6*C*). Changes in gene expression that were observed in response to LPS are transient, as the expression levels of *Gbp2*, *Gbp5*, *Mafa*, and *Slc2a2* return to baseline within 24 h of LPS administration in both βIl1r1KO and littermate control animals (Fig. 6, *B* and *C*). Cytokines in addition to IL-1 likely contribute to the repression of identity gene expression, as loss of IL-1 signaling receptor in βIl1r1KO mice does not modify the effects of LPS administration on islet *Mafa* and *Slc2a2* mRNA levels. TNF is one candidate cytokine that represses the expression of endocrine cell identity genes (31, 32), and like IL-1, TNF represses β-cell identity gene expression (*Mafa* and *Slc2a2*) following 6 h cytokine treatment in islets from C57BL/6J mice (Fig. 6*D*). Repression of these genes in response to TNF is independent of β-cell IL-1R signaling, as shown in Figure 6*E*, indicating these are likely direct effects of TNF on β-cells rather than secondary effects mediated by IL-1.

### Dose-dependent effects of LPS on transcriptional changes in islets

**Figure 7:**
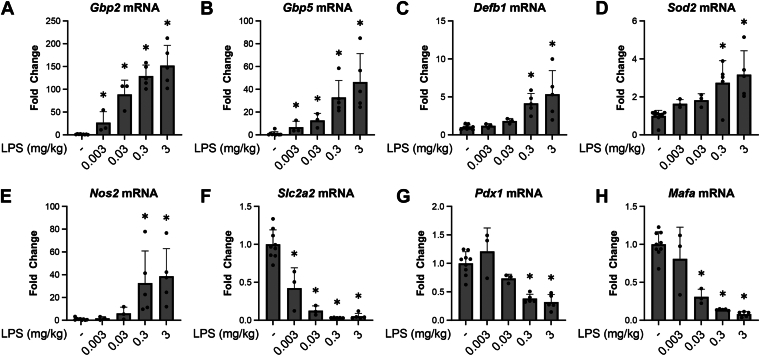
**Dose-related actions of LPS administration on gene expression in islets.** Islets were harvested 6 h post IP administration of 0.003 to 3 mg/kg LPS or endotoxin-free physiological saline to C57BL/6J mice, mRNA was isolated, and (A-E) induced genes *Gpb2*, *Gbp5*, *Defb1*, *Sod2*, and *Nos2* and (F-H) genes with decreased expression *Slc2a2*, *Pdx1*, and *Mafa* were evaluated by qRT-PCR. Results are the average ± SD of 3 to 5 mice per group. Statistical significance calculated by one-way ANOVA (∗*p* < 0.05 *versus* untreated control; ns = not significant) is indicated. Defb1, beta-defensin 1; Gbp2, guanylate binding protein 2; Gbp5, guanylate binding protein 5; LPS, lipopolysaccharide; Sod2, superoxide dismutase 2.

LPS is an extensively characterized PAMP that has been used to mimic responses to bacterial infection. When delivered at low doses (0.33 mg/kg), LPS induces a transient illness behavior characterized by early (3–6 h) loss of social behavior that returns to baseline by 24 h (15). We show (Figs. 3 and 4) that changes in islet cell gene expression follow a similar time-course, with early changes at 3 to 6 h followed by return to control levels by 24 h. When administered to mice at doses ≥10-fold higher, LPS has been used as a model of systemic inflammatory response syndrome, as it mimics the effects of high levels of pro-inflammatory cytokines that are released during sepsis (17). LPS was given at 0.003 to 3 mg/kg to assess the dose-dependent effects on islet gene expression 6 h post administration to C57BL/6J mice. Similar responses were observed when comparing a dose of 0.33 mg/kg (mimics mild bacteremia) *versus* 3 mg/kg (dose used in sepsis models) on islet gene expression, as the changes in mRNA accumulation of *Gbp2*, *Gbp5*, *Defb1*, *Sod2*, *Nos2*, *Slc2a2*, *Pdx1*, and *Mafa* appear to plateau between 0.3 to 3 mg/kg LPS (Fig. 7). We were surprised that several genes are differentially expressed in islets in response to LPS doses as low as 0.003 mg/kg. These changes include increases in antiviral genes *Gbp2* and *Gbp5*, and decreases in *Slc2a2* mRNA levels. These results suggest that endocrine cells are highly responsive to increases in serum levels of cytokines as LPS doses as low as 0.003 mg/kg are capable of modifying gene expression in endocrine cells. Near-maximal changes are observed at levels of LPS that are associated with mild bacteremia (0.33 mg/kg) as this response is not dramatically increased using septic doses of LPS (3 mg/kg).

## Discussion

The *in vitro* treatment of islets with cytokines such as IL-1 results in an early potentiation (∼30% at 3 h) followed by the time-dependent inhibition of insulin secretion that is first apparent 5 to 8 h after cytokine addition and is nearly complete following an 18 h exposure (3). Prolonged incubation of isolated islets for 36 h or longer results in irreversible inhibition of insulin secretion and islet degeneration (10). Based on the results of these *in vitro* studies, it has been hypothesized that cytokines contribute to the loss of β-cell mass during the development of T1D. However, the inhibitory actions of cytokines on insulin secretion can be reversed by either washing the cytokine away (11) or inhibiting iNOS (9), and it is only after prolonged exposures of 36 h or longer that the actions of cytokines become irreversible and islet cell death is observed (9, 10). The ability of β-cells to recover metabolic and secretory function suggested to us that cytokine signaling in the endocrine compartment may play physiological roles. In support of this hypothesis, scRNA-seq analysis of isolated mouse and human islets identify that one early (6 h) transcriptional response of endocrine cells to IL-1 and IL-1 + IFN-γ is the enhanced expression of antipathogen genes. Most studies have focused on the inhibitory and destructive actions of cytokines using *in vitro* approaches where islets are exposed to cytokines for prolonged periods of time (up to days) prior to evaluating the functional changes. In this study, we have developed an *in vivo* approach to evaluate the islet response to endogenously produced cytokines, with a focus on gene products known to influence β-cell function *in vitro*.

**Figure 8:**
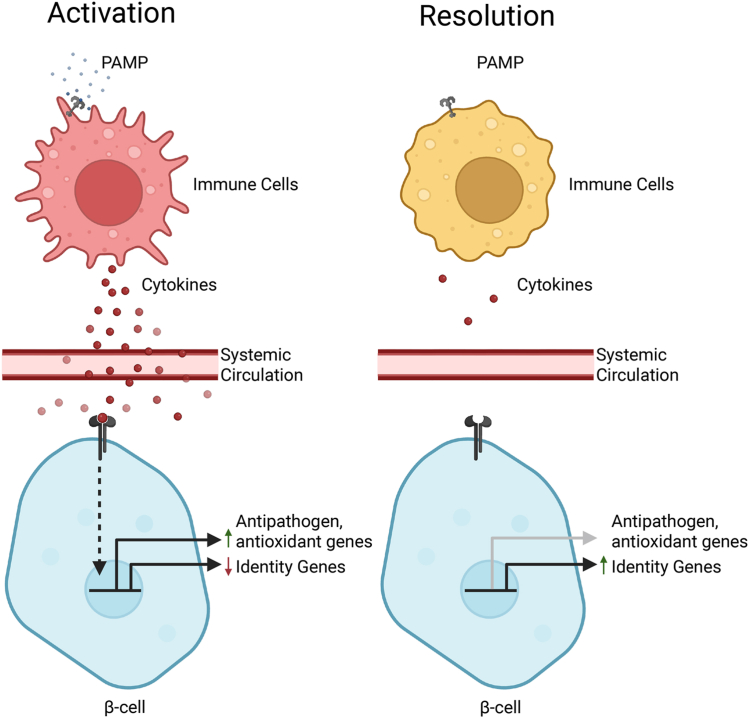
**Model.** Following stimulation by pathogen-associated molecular pattern, activated innate immune cells respond by secreting cytokines that travel through the systemic circulation to reach the pancreatic islet resulting in transient expression of antipathogen genes and repression of identity genes that resolves following clearance of the inciting pathogen-associated molecular pattern.

To address this goal, the well-characterized bacterial PAMP, LPS, was used to activate the innate immune system and stimulate the endogenous production of cytokines in mice. Within 3 to 6 h of LPS administration, we observed an increase in the expression of antiviral, antibacterial, antioxidant, and proinflammatory genes in islets. We also observed a decrease in the mRNA accumulation of β-cell identity factors. Consistent with the actions of cytokines being reversible *in vitro* (9, 10), the changes in gene expression we observe in islets harvested from mice 6 h post LPS administration are not observed in islets isolated 24 h post LPS administration (Fig. 8). LPS is not directly responsible for initiating a new transcriptional program in endocrine cells, as this PAMP fails to modify gene expression in primary β-cells and insulinoma cells (Fig. 2). Importantly, within minutes of PAMP exposure, macrophages produce proinflammatory cytokines such as IL-1 and TNF (33, 34), and our data suggest that it is these soluble mediators that direct the transcriptional response in islet endocrine cells. IL-1 plays a primary role in this response, as islets from mice harboring a β-cell selective deletion of the IL-1 signaling receptor fail to respond normally to LPS administration. Soluble mediators in addition to IL-1 also influence these responses. For example, interferons stimulate guanylate binding protein expression (35) and TNF represses β-cell identity gene expression (32, 36). In fact, the loss of IL-1-signaling in β-cells does not prevent the loss of identity genes in response to LPS administration, suggesting that additional cytokines, such as TNF, likely contribute to transcriptional changes in endocrine cell gene expression in response to LPS administration.

The transcriptional changes we observed in islets in response to LPS administration are consistent with the changes we observed in endocrine cells following a 6 h incubation of isolated mouse islets with IL-1 and IL-1+ IFN-γ (12). These exciting results provide evidence that the initial responses of endocrine cells to cytokines observed *in vitro* also occur *in vivo* in response to innate immune activation. In fact, LPS administration has long been known to stimulate a rapid drop in blood glucose levels (4 h) (16), which corresponds to the time-dependent increase (2–3 h) in insulin secretion that is observed early in the treatment of isolated islets with IL-1 (26). In contrast, when islets are cultured for 24 or 48 h *in vitro*, cytokine-stimulated gene expression remains elevated (37), yet *in vivo*, 24 h post LPS administration islet cell gene expression returns to basal levels. These differences likely reflect the transient increase in serum cytokine levels in response to LPS administration (17) compared with the continuous presence of cytokine in isolated islet cultures. Importantly, LPS administration does not cause diabetes, and diabetes is not a typical outcome of sepsis. This likely reflects the absence of direct β-cell responses to this PAMP, the transient nature of cytokine production by immune cells following activation, the maximal response of endocrine cells to low levels of LPS that we observe, and yet-to-be-defined regulatory mechanisms that may also control endocrine cell gene expression *in vivo* following innate immune activation.

As described above, LPS administration to rodents has been shown to induce a rapid early hypoglycemia that is associated with enhanced insulin secretion (38, 39). We have extended these original observations to show that 24 h post LPS administration, insulin secretion is elevated, and glucose tolerance is enhanced as compared to control mice. At 24 h post administration of LPS, there is a loss of β-cell identity genes that occurs under conditions of enhanced insulin secretion, suggesting that the absence of identity gene expression does not attenuate β-cell function. While these effects on β-cell function may be surprising, it is known that *in vitro* treatment of islets with IL-1 results in the loss of β-cell identity gene expression and an inhibition of glucose stimulated insulin secretion (6, 7, 13). Inhibitors of iNOS prevent cytokine-mediated inhibition of insulin secretion but do not modify cytokine-mediated loss of identity gene expression (6, 7, 13). In addition, the stimulatory actions of LPS on insulin secretion 24 h post administration occur under conditions in which iNOS is expressed in insulin containing cells. This observation may also be surprising, as nitric oxide, the product of iNOS, is known to mediate the inhibitory actions of cytokines on β-cell function (5, 28). We hypothesize that the levels of nitric oxide produced in islets in response to LPS administration do not reach the levels necessary to inhibit mitochondrial function and insulin secretion. We have shown that at concentrations in excess of 0.7 to 1 μM, nitric oxide inhibits mitochondrial oxidation, decreases β-cell ATP levels 8 to 10 fold, and inhibits insulin secretion (4, 40, 41). At concentrations below ∼0.7 μM, this free radical does not inhibit mitochondrial function, but enhances the expression of protective genes (4, 40, 41). It is also challenging to compare the results of *in vivo* studies with *in vitro* experiments. Nitric oxide is an uncharged free radical that is freely diffusible across membranes and is known to bind to heme containing moieties in cells, such as hemoglobin in red blood cells. *In vitro*, nitric oxide is statically produced by islets in a dish, while *in vivo* islet blood flow likely reduces the local effective concentrations.

Islet blood flow also contributes to the responses observed following LPS administration. Although islets of Langerhans make up only approximately 1% of the wet weight of the pancreas, they receive up to 20% of the pancreatic blood flow (42), allowing them to rapidly detect changes in blood glucose and secrete endocrine hormones in response. As islet endocrine cells sense blood glucose levels, they are exposed to all components of the blood, including circulating cytokines that are elevated in response to pathogens or PAMPs. As we show in this study, β-cells sense serum cytokines like IL-1 and respond with transcriptional changes in the endocrine pancreas that include increased expression of antipathogen and inflammatory genes and decreased expression of identity factors. Despite reduced expression of β-cell identity proteins 24 h after LPS administration, insulin secretion is further increased, rather than diminished, in these animals in response to glucose challenge.

If cytokines play a primary role in the development of T1D by inducing the loss of β-cell mass, one could expect cytokine-mediated β-cell damage during any infection or immunological event that increases serum cytokine levels. In contrast, we hypothesize that cytokine signaling in the endocrine compartment is physiologically relevant and functions to initiate β-cell autonomous defense pathways that include decreases in identity gene expression, the induction of genes whose products function in antipathogen defense, and the induction of inflammatory genes such as iNOS that produce mediators such as nitric oxide that limit insulin secretion but also attenuate β-cell apoptosis (43) and virus replication (44, 45). Environmental factors such as infection (viral and bacterial) have been proposed as precipitating events leading to the induction of T1D pathogenesis (46, 47), and cytokines produced in response to infection may contribute to disease pathogenesis (48). Our findings in this study provide evidence that cytokine signaling in the endocrine pancreas may play an important protective role, activating defense pathways that contribute to the protection of β-cells from environmental insults, and it is when these pathways fail to respond normally that disease initiation occurs. If correct, this would represent a paradigm shift in our understanding of pathways contributing to T1D development and stimulate new approaches to address the mechanisms of pathogenesis of a disease that has remained elusive for many decades.

## Experimental procedures

### Animals and cells

Male C57BL/6J mice (RRID:IMSR_JAX:000664) were purchased from The Jackson Laboratory and housed in the MCW Biomedical Resource Center with 14:10 h light/dark cycle. Mice expressing Cre in the endogenous Ins1 locus (B6(Cg)-Ins1^tm1.1(cre)Thor^/J; RRID:IMSR_JAX:026801) (30) were bred to mice containing a floxed Il1r1 gene (Il1r1^flox/flox^) with loxP sites positioned to flank exons 3 and 4 (B6.129(Cg)-Il1r1^tm1.1Rbl^/J; The Jackson Laboratory; RRID:IMSR_JAX:028398 (29), to generate mice with β-cell conditional deletion of Il1r1 (βIl1r1KO). Animal breeding and experimental procedures were approved by the Institutional Animal Care and Use Committee at the Medical College of Wisconsin. INS 832/13 cells were obtained from Dr Christopher Newgard (Duke University).

### Islet isolation and islet/cell culture

Islets were isolated from mice using methods previously described (49), and either immediately lysed for qRT-PCR analysis (*in vivo* experiments) or cultured at 37 °C and 5% CO_2_ in Connaught Medical Research Laboratories (CMRL) 1066 culture media (Thermo Fisher Scientific) containing 5.5 mM glucose and supplemented with 10% heat-inactivated fetal bovine serum (FBS; HyClone), penicillin and streptomycin (Thermo Fisher Scientific), and L-glutamine (Thermo Fisher Scientific). INS832/13 cells were cultured in Roswell Park (RPMI) 1640 medium (Thermo Fisher Scientific) supplemented with 10% heat-inactivated FBS, 2 mM L-glutamine 1 mM sodium pyruvate, 10 mM Hepes, and 50 μM 2-mercaptoethanol (Thermo Fisher Scientific).

### Reagents

Lipopolysaccharide (LPS) from *Escherichia coli* O111:B4 (S-form) at a concentration of 1 mg/ml (Cat. # IAX-100-012-M001) and endotoxin-free physiological saline (Cat. # IAX-900-003-L010) were purchased from Innaxon for IP injection. Human recombinant IL-1β, murine IFN-γ, murine TNF-α, and human IL-1RA recombinant protein (Cat. # 200-01RA) were purchased from PeproTech.

### Islet dispersion and FACS purification

B6(Cg)-Ins1^tm1.1(cre)Thor^/J mice were bred to mice expressing the dual-color fluorescent Cre reporter ROSA26mTmG (B6.129(cg)-*Gt(ROSA)26Sor*^*tm4(ACTB-tdTomato,-EGFP)Luo*^/J) purchased from The Jackson Laboratory (RRID:IMSR_JAX:007676). Cells express membrane targeted tandem dimer Tomato (mT) before Cre-mediated excision or membrane targeted green fluorescent protein (mG) after Cre recombination. Islets, isolated from *Ins1*^*Cre/+*^*; ROSA26*^*mTmG/+*^ mice, were dispersed using 0.1% trypsin prior to FACS to obtain purified GFP^+^ and Tomato^+^ cell populations as previously described (50).

### RNA isolation and real-time PCR analysis

Total RNA was isolated from islets, FACS-purified cell populations, or INS832/13 cells using Qiagen RNeasy Mini Kit and DNA digestion was performed using TURBO DNA-free Kit (Invitrogen). Maxima H Minus reverse transcriptase (Thermo Fisher Scientific) and oligo(dT)_20_ primers were used to perform first-strand cDNA synthesis. SsoFast EvaGreen supermix (Bio-Rad) and primers purchased from Integrated DNA Technologies (Table S1) were used to perform quantitative PCR with a Bio-Rad CFX Duet Real-Time PCR System (Bio-Rad). Gene expression was normalized to *Gapdh* using the comparative C_t_ method (51).

### Immunofluorescent imaging analysis

Mouse pancreases were fixed in formalin overnight, paraffin embedded and sectioned at a thickness of 4 μm. Slides were de-waxed, subjected to antigen retrieval (100 °C in 10 mM trisodium citrate pH 6.0/0.05% Tween-20 for 20 min) and allowed to cool at room temperature for 30 min. Sections were permeabilized for 30 min (0.1% Triton X-100 in PBS) and blocked using 1% BSA in PBS-T (0.2% Tween-20) for 1 h at room temperature. Sections were incubated in primary antibody diluted in 1% BSA in PBS-T overnight at 4 °C. The following antibodies were used: guinea pig anti-insulin (1:1000; Dako Cytomation), rabbit anti-GBP2 (1:500; Proteintech), rabbit anti-iNOS (1:200, Cayman Chemical), rabbit anti-PDX1 (D59H3) (1:1000, Cell Signaling Technology), rabbit anti-glucose transporter GLUT2 (1:200, Abcam), donkey anti-guinea pig conjugated to Alexa Fluor 488 (1:1000; Thermo Fisher Scientific), and donkey anti-rabbit conjugated to Cy3 (1:1000; Thermo Fisher Scientific). Slides were probed with DAPI (1:8000; D21490; Thermo Fisher Scientific) and mounted with ProLong gold antifade mountant (Thermo Fisher Scientific) and sealed with a coverslip. Slides were imaged with a Nikon Spinning Disk Confocal (CSU-W1) microscope using Plan Fluor 40*×* DIC M objective. Images were captured using Nikon Elements software (Nikon Elements Advanced Research v 6; https://www.microscope.healthcare.nikon.com/en_AOM/products/software/nis-elements/nis-elements-advanced-research) and analyzed using ImageJ software (http://imagej.net/ij/download.html; National Institutes of Health).

### Histology imaging analysis

Pancreas sections were formalin fixed, paraffin embedded, sectioned at a thickness of 4 μm, and subjected to H&E stain. Assessment of histologically stained sections was performed using a Nikon NiE upright microscope using a 10× Plan Apo λD 0.45 NA objective for the pancreas overview and a 40× Plan Apo λD 0.95 NA objective for the single images of islets. The NiE is equipped with a 5.9-megapixel color camera (Nikon DSFi3), a fully motorized Prior stage and Nikon’s z-controller. Images were captured using Nikon Elements software (Nikon Elements Advanced Research v 6). CellProfiler v4.2 was used to quantify mean fluorescent intensity of iNOS, GBP2, PDX1, or GLUT2 per insulin-positive cell.

### Western blot analysis

Cells were washed with PBS and lysed in Laemmli buffer prior to separation of proteins by SDS-PAGE and Western blot analysis using methods described previously (52). The following antibodies were used: lamin B (1 μg/ml, Proteintech), IκBα (1:1000, Cell Signaling Technology), mouse anti-GAPDH (1:10,000, Thermo Fisher Scientific), and rabbit anti-iNOS (1:1000, Cayman Chemical). Antigen was detected by chemiluminescence (53) using HRP-conjugated donkey anti-rabbit (1:10,000) or donkey anti-mouse (1:10,000) secondary antibodies from Jackson ImmunoResearch Laboratories.

### Serum cytokine analysis

Terminal blood collection was performed by percutaneous cardiocentesis immediately following CO_2_ asphyxiation. Serum was collected following centrifugation in Microvette 300 Serum CAT tubes (Cat. # 20.1308.100, Sarsteedt Inc.) and cytokine concentrations were measured using the LEGENDplex Mouse Anti-Virus Response Panel (Cat. # 740622, BioLegend) according to manufacturer instructions.

### Oral glucose tolerance test

Glucose tolerance testing was performed 24 h after IP injection of saline or 0.33 mg/kg LPS. Food was removed 4 h prior to the test, which was initiated by delivery of 2 g glucose/kg body weight *via* oral gavage. Blood was sampled from a tail vein; glucose was measured using a OneTouch UltraMini glucometer, and whole blood insulin was measured using the Ultra Sensitive Mouse Insulin ELISA kit (#90080) from CrystalChem using the protocol modifications described by Small *et al.* (54).

### Statistics

Statistical comparisons are made between two groups with Student's *t* test or three or more independent conditions with ANOVA and Tukey’s *post hoc* analysis. Significant differences are indicated (∗*p* < 0.05).

## Data availability

Sequencing data shown in this publication was previously published (Stancill *et al.*, 2021) and is available in the National Center for Biotechnology Information (NCBI) Gene Expression Omnibus database under accession number GSE156175.

## Supporting information

This article contains supporting information.

## Conflict of interest

The authors declare that they have no conflicts of interest with the contents ofthis article.
