# Supplementary material for: Lipopolysaccharide-induced cytokine signaling activates a temporal innate defense program and represses pancreatic β-cell identity
Source: J Biol Chem. 2025 Oct 13;301(12):110811. doi: 10.1016/j.jbc.2025.110811 (PMC12663513; doi:10.1016/j.jbc.2025.110811)
Supplement: Supporting Figures Tables [file mmc1.pdf]

Supplementary Material

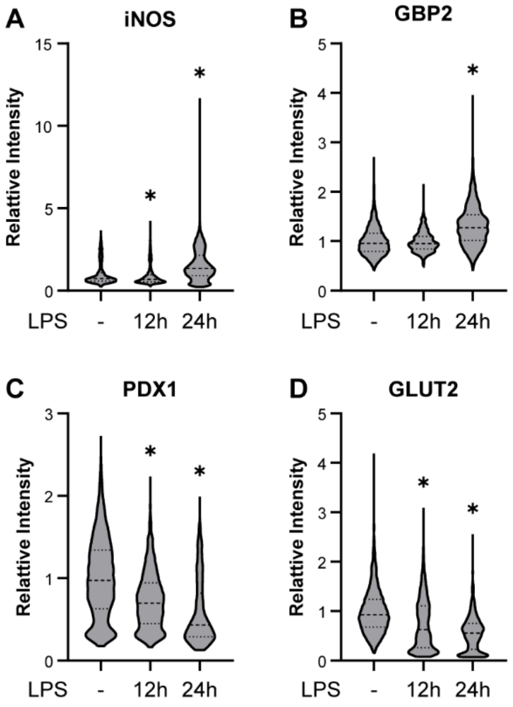

Fig. S1. Quantification of the intensity of (A) iNOS, (B) GBP2, (C) PDX1, and (D) GLUT2 staining per β-cell in pancreatic sections collected from mice 12 h or 24h after intraperitoneal LPS administration (0.33 mg/kg). The quantified data represent 3 replicates with an average of N = 23 islets per condition. Median and interquartile range are shown with dashed lines and expression that is significantly changed from the saline control is indicated (*p < 0.05).

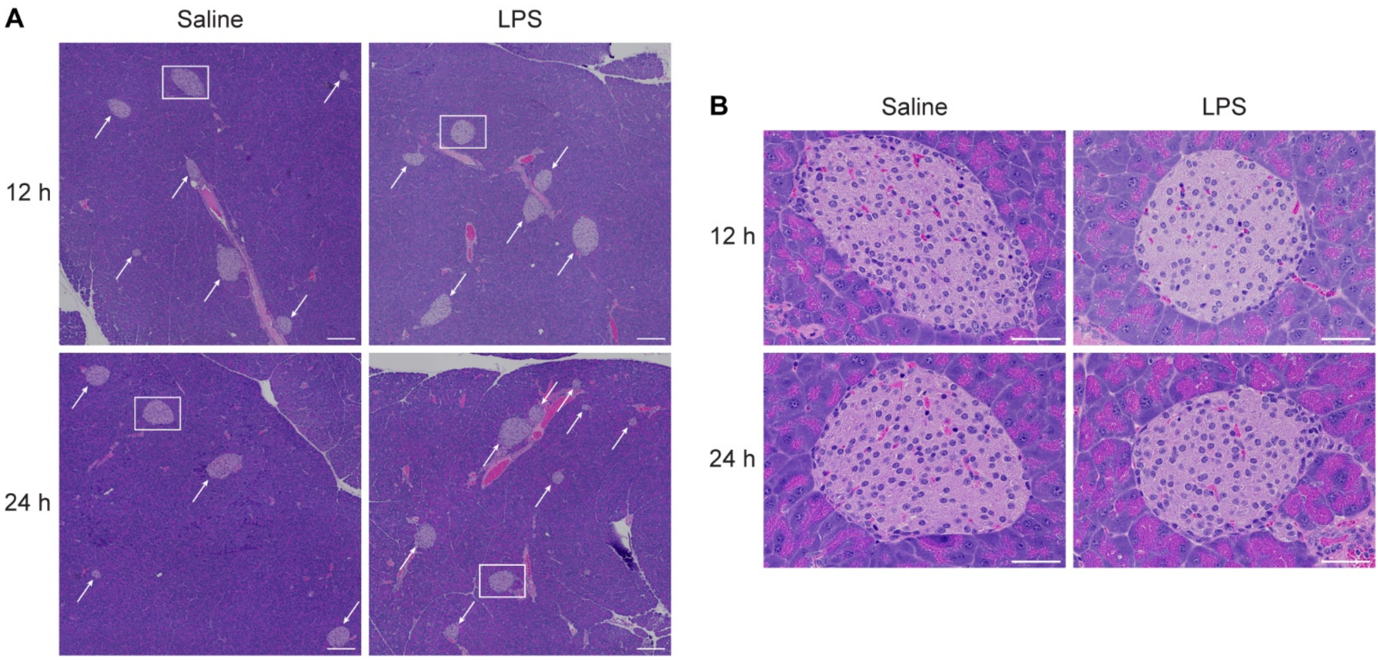

Fig. S2. Representative images from pancreatic sections from mice administered saline or LPS (12h or 24h) subjected to hematoxylin and eosin stain. (A) Scale bar = 200 μm. (B) Scale bar = 50 μm.

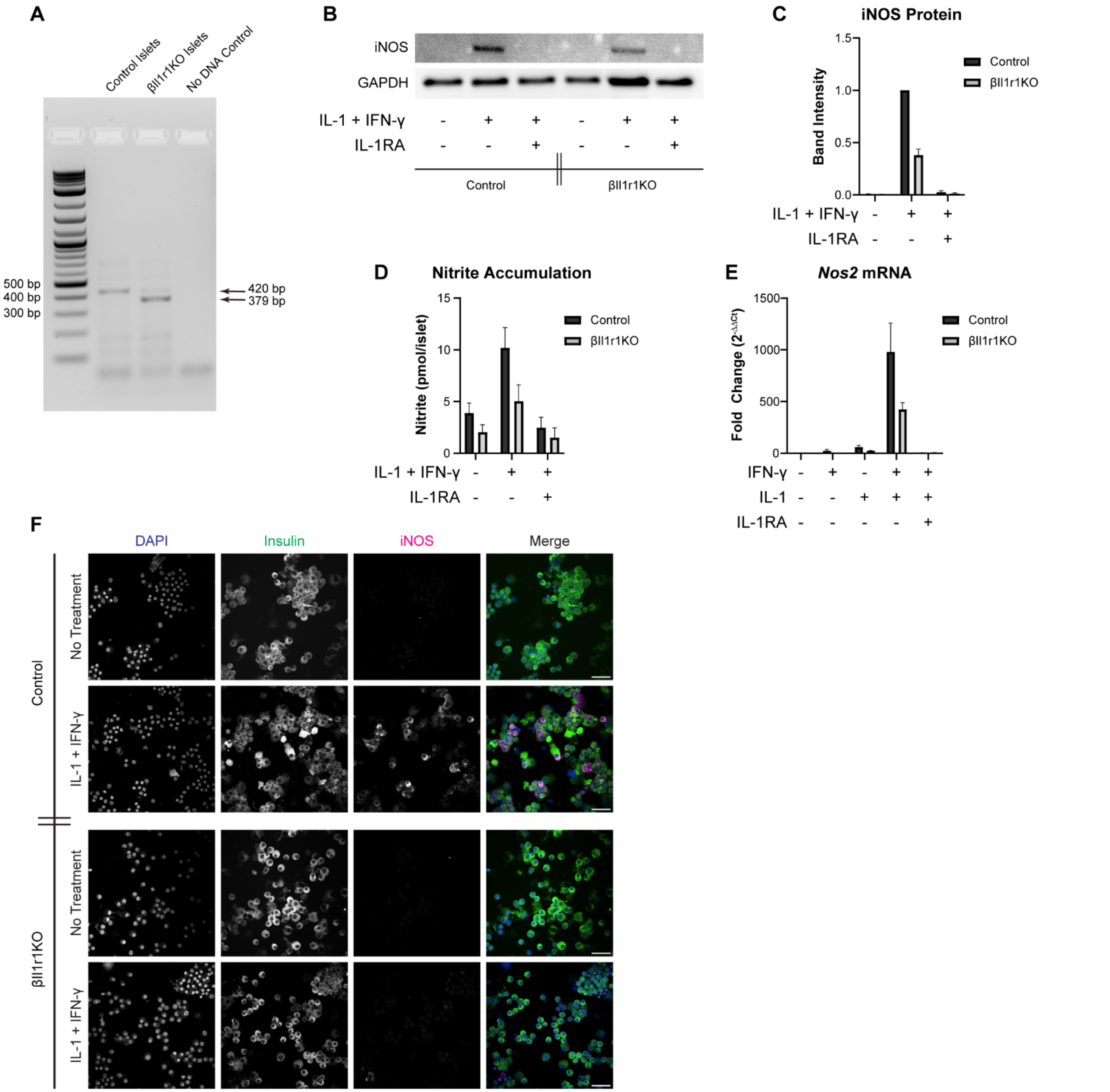

Fig. S3. (A) Genomic DNA from βIl1r1KO (Il1r11$^{fl/fl}$; Ins1$^{Cre/+}$) and littermate control animals (Il1r11$^{fl/fl}$; Ins1$^{+/+}$). (B-C) iNOS protein expression, (D) nitrite accumulation, and (E) Nos2 mRNA in mouse islets treated in vitro with IL-1 + IFN-γ with or without IL-1 receptor antagonist protein (IL-1RA) (average ± SEM). (F) Representative images of dispersed islets from βIl1r1KO or littermate control animals treated with cytokines for 40 h and probed for DAPI, insulin, and iNOS.

Gene	Species	Forward Primer (5' → 3')	Reverse Primer (5' → 3')
Defb1	m,r	TTCACATCCTCTCTGCACTCTG	GTCCAAGACTTGTGAGAATGCC
Gapdh	m,r	GACATCAAGAAGGTGGTGAAGC	TCCAGGGTTTCTTACTCCTTGG
Gbp2	m	TGTGGGCTTCTTTCCAACCT	CCAGTTTGCTAAGTTGCTTCC
Gbp2	r	TGTGGGCTTCTTTCCAACCT	CCAGTTTGCAGAGTTGCTTCC
Gbp5	m,r	GGGGATCTGGATGTGGTGTG	GCAATAGGTCGATAGCCCCC
Il1r1	m	GCACGCCCAGGAGAATATGA	AGAGGACACTTGCGAATATCAA
Mafa	m,r	CCTGTAGAGGAAGCCGAGGAA	CCTCCCCCAGTCGAGTATAGC
Nos2	m,r	CGAGACTTCTGTGACACACAGC	CATCTCCTGGTGGAACACAGGG
Pdx1	m,r	GACCTTTCCCGAATGGAACC	TTTCCACGCGTGAGCTTTG
Slc2a2	m,r	TTCAGCAACTGGGTCTGCAA	TGAAGATCAGGACCACCCCA
Sod2	m,r	TGGTGGAGAACCCAAAGGAGAG	AGAGCAGGCGGCAATCTGTAAG

Table S1. PCR primer sequences.
